# The Dependence of Electrode Impedance on the Number of Performed EEG Examinations

**DOI:** 10.3390/s19112608

**Published:** 2019-06-08

**Authors:** Joanna Górecka, Przemysław Makiewicz

**Affiliations:** Department of Systems, Signals and Electronic Engineering, Faculty of Electrical Engineering, West Pomeranian University of Technology Szczecin, 70-313 Szczecin, Poland; przemyslaw.makiewicz@zut.edu.pl

**Keywords:** electroencephalography (EEG), electrochemical impedance spectroscopy (EIS), reusable electrodes

## Abstract

In clinical practice, it is recommended to employ reusable electrodes for the registration of brain waves. Before registering EEG signals, the EEG technician checks the condition of all the electrodes, i.e., the occurrence of mechanical damage and the color of the electrode coating. It should be noticed that there is still no information on the permissible number of EEG examinations performed with one set of electrodes. After placement of the electrodes on the patient’s head, the scalp–electrode impedance is measured with the use of EEG equipment. When the scalp–electrode impedance achieves a value above 5 kΩ, it is necessary to replace the given electrode or to re-execute skin abrasion. The Electrochemical Impedance Spectroscopy (EIS) method was used in order to estimate the permissible number of EEG examinations performed with one set of electrodes. Ten new reusable electrodes were tested. Then, the tests were repeated after subsequent uses of those electrodes. The conducted tests led us to the conclusion that the permissible number of examinations performed with one set of electrodes is up to twenty except for the gold electrodes for which it is up to ten. Furthermore, the use of the EIS method revealed variability of impedance in the case of new electrodes.

## 1. Introduction

Electroencephalography as a functional method for studying brain electrical activity is still the basic diagnostic tool in neurology. In addition to being used in the diagnosis and treatment of epilepsy, EEG records are used to detect toxic-metabolic disorders and other neurological disorders characterized by confusion and disturbances of consciousness [1]. The registration of EEG signals has also been used in the research of sleep and its disorders [2] (pp. 199–211). Currently, digital EEG equipment is used, therefore, the quality of registered signals is primarily influenced by the selection of suitable electrodes, electrode pastes and gels, the skin properties of the subject, and the preparation of the skin [2] (pp. 19–27), [3,4,5]. 

The frequency properties of the various types of reusable electrodes, conducting pastes and gels are widely considered in [3,6,7]. It should be noticed that any metal (i.e., gold, silver, stainless steel, or tin) can be used for electrodes [6]. Currently, electrodes are proposed that do not require additional usage of a conductive paste or gel [8,9,10,11,12,13]. In clinical practice, not all proposed electrodes can be used to record EEG signals [3,6]. Pure silver electrodes are distinguished by the possibility of their polarization, but their resistance for the slow EEG frequencies (i.e., below 10 Hz) rises exponentially, and it leads to a distortion of the EEG signal [6]. For that reason, they are preferred for measurements of evoked potentials. In clinical practice, it is recommended to use gold or silver/silver chloride cup electrodes in the EEG frequency range, i.e., (0.5–70) Hz [14,15]. Gold and silver/silver chloride cup electrodes usually consist of a silver cup coated with either gold or silver chloride alloy. The thickness of the coating depends on the manufacturer and varies within a range of 2–3 μm. The material from which gold and silver/silver chloride cup electrodes are made prevents the generation of additional noises and, in comparison with electrodes made of other materials, does not significantly distort the signal in the recording range of electroencephalograms [6]. Silver/silver chloride electrodes are very popular because of the high stability of their electrical properties [5,6]. This stability is achieved with the properties of an electrodeposited coating that can be easily removed by abrasion. In addition, the surface layer of those electrodes is photosensitive. Hence, during photic stimulation, such electrodes should be shaded from the changing intensity light [6]. The other problem is related to recording of the EEG signal in the range of slow waves, i.e., below 0.5 Hz. In [3] it has been shown that only silver/silver chloride electrodes should be used for recording the slow EEG potentials because, besides the lowest resistance and the superior low-frequency noise, in comparison to the gold electrodes they have much better DC-stability. 

In clinical practice, the scalp-electrode impedance is checked before each EEG examination. In digital EEG equipment, impedances up to 10 kΩ are usually acceptable, but values below 5 kΩ are still recommended [14,15]. Nevertheless, impedance lower than 100 Ω is unacceptable, as it often indicates a shunt or short circuit related to a salt bridge on the scalp [7]. In order to reduce the impact of disturbances and obtain a scalp–electrode impedance lower than 5 kΩ skin abrasion is still required [14,15], but in some cases it is not recommended [4]. The additional use of electrode pastes and gels also reduces the scalp-electrode impedance [3,7,14,15]. Maintaining the electrodes in a state of adequate purity is also required to achieve the desired values of impedance. 

In clinical practice, achieving the impedance of the silver/silver chloride or gold electrodes below 5 kΩ is not so difficult. It is important to obtain the same value of the scalp-electrode impedance for each of the electrodes used in a given study, especially for registration of low-voltage EEG signals. According to the recommendations presented in [14,15], it is enough to use a set of electrodes made of the same material, cable length, and diameter. It is assumed that electrodes of the same type and from the same manufacturer should have the same frequency parameters in a range of 0.5–70 Hz, while small differences in values of the scalp–electrode impedance may result from the skin properties of the subject (e.g., seborrheic or atopic dermatitis, lipomas) or preparation of the skin.

Another problem is to obtain correct and uniform value of the scalp–electrode impedance of the selected set of electrodes, which is associated with their repeated use. In work [3], it was noted that significant differences in performance of new and used silver electrodes occur. New silver electrodes were unstable and had large offset voltages whereas the used silver electrodes approached the behavior of silver chloride electrodes. This change was a result of spontaneous formation of a silver chloride layer on the surface of electrodes. 

In the literature, to the best knowledge of the authors of this article, there is no information on the permissible number of EEG examinations performed with one set of silver/silver chloride or gold electrodes. This paper presents the analysis of changes in the impedance value in the frequency range of brain waves of electrodes used in routine EEG examination, including the number of tests performed. 

## 2. Materials and Methods

In order to investigate the influence of the amount of performed routine EEG examinations on the electrode impedance, four types of reusable electrodes were tested: gold (FS-E5GH-60; Genuine Grass), silver (SC12-626-GVB), silver/silver chloride (SC22-626-GVB), and sintered bridge electrodes with gold pin (SBELEK7-GVB). Cup and sintered bridge electrodes with 10 mm diameter were used. The length of the wires of the gold electrodes was 152 cm and 150 cm for all other electrodes. All EEG examinations were carried out on one of the authors of this paper. Ten electrodes from each type were examined. All electrodes were tested before the first use, after the first, the fifth, the tenth and the twentieth uses. In addition, three types of electrodes were used successively as a reference electrode, i.e., gold, silver, and silver/silver chloride electrodes. In the case of sintered bridge electrodes, silver/silver chloride reference electrodes were used. It should be noticed that for every EEG examination a new reference electrode was used. All EEG examinations were collected at a 200 Hz sampling rate from the Cz channel in bandwidth: (0.1–100) Hz. According to the recommendations presented in [2] (pp. 19–27) a reference electrode has been placed on the right earlobe. The unipolar montage was used for these experiments. Additionally, a disposable silver/silver chloride electrode with snap button (CDES003545-GVB) was used as a ground. This electrode was attached to the forehead.

After cleansing scalp and right earlobe with NUPREP preparation gel and ECOLAB Skinsept Pur, all cup electrodes were applied to the head with the TEN20 conductive EEG paste. Before measurements, the sintered bridge electrodes were soaked in 0.9% NaCl solution for 24 h. In order to place them on head, the EEG cap was used. Previously, they were covered with the conducting gel-ECI Electro-Gel. Then, to confirm the value of scalp-electrode impedance below 5 kΩ for each of the electrodes, Grass Comet-PLUS EEG/PSG amplifier system was used. After the duration of the routine EEG examination (i.e., 20 min) without activation procedures (i.e., photic stimulation or hyperventilation), all electrodes were cleaned according to the recommendations of manufacturers and prepared for further tests. 

**Figure 1:**
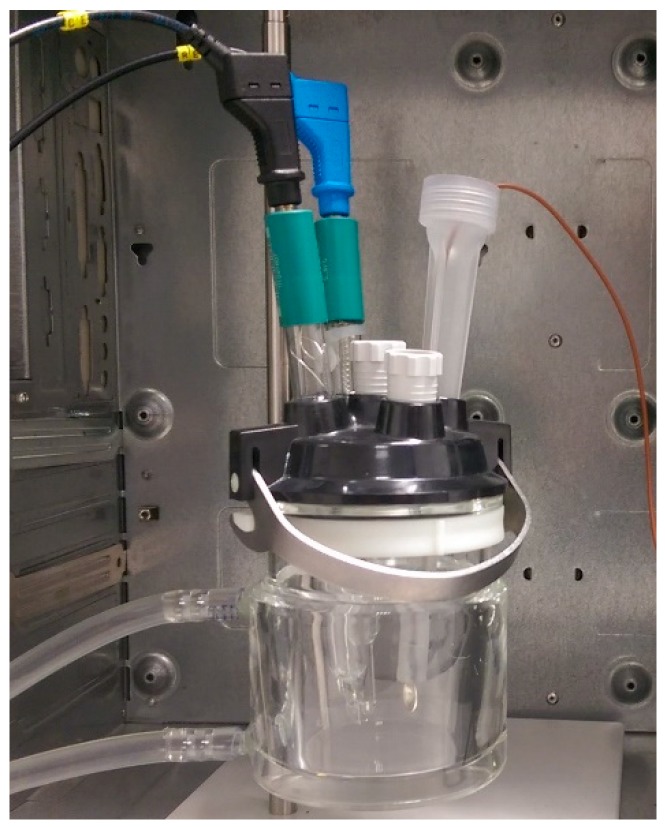
An electrochemical cell with a three-electrode setup.

The Electrochemical Impedance Spectroscopy (EIS) method was applied to check the changes of impedance of electrodes related to the number of performed EEG tests. All electrodes were tested in a frequency range of 0.1–100 Hz and in a temperature of 37 degrees. The choice of temperature was based on normal body temperature for a healthy subject. All measurements were performed with use of the Autolab PGSTAT302N platform with an additional module for impedance measurements-FRA32M. It should be noticed that for each tested frequency a single sinusoidal signal with amplitude of 200 μV was applied to every EEG electrode. An electrochemical cell (Methrom, product No. 6.1418.250) connected with Julabo F12-ED thermostat and additional thermal insulation-Armaflex ACE-32-99 were used to ensure repeatable conditions. A complete electrochemical cell used for our experiments is shown in Figure 1.

In order to prevent any noises, all measurements were performed within a Faraday cage with a three-electrode setup, i.e., examined EEG electrode, reference (silver/silver chloride electrode Metrohm 6.0733.100), and counter (platinium electrode Metrohm 3.109.0790) electrodes. Potential occurring between these electrodes is used as reference point for a signal applied to the counter electrode and the examined electrode. For the cup electrodes and the sintered bridge electrodes, a TEN20 conductive EEG paste and ECI Electro-Gel were placed in the electrochemical cell, respectively. Electrodes used as a reference electrode in EEG examinations have not been tested with the use of EIS.

## 3. Results

**Figure 2:**
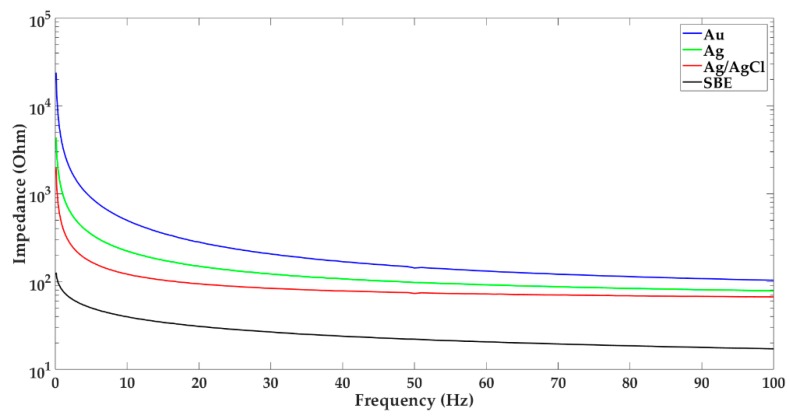
Impedance characteristic of the following new electrodes: gold (Au), silver (Ag), silver/silver chloride (Ag/AgCl), and sintered bridge electrode with gold pin.

In Figure 2, impedance characteristics of reusable electrodes commonly used in clinical practice are shown. Mean values of impedance were calculated based on the measurements of ten electrodes of each type.

The presented results confirm the generally known fact that the impedance decreases with increasing frequency [6]. However, the sintered bridge electrodes are characterized by the smallest impedance in the entire EEG frequency range in contrast to the gold electrodes, whose impedance is the largest. It is worth adding that none of new, tested electrodes in the range of slow waves has obtained a linear characteristic. 

**Figure 3:**
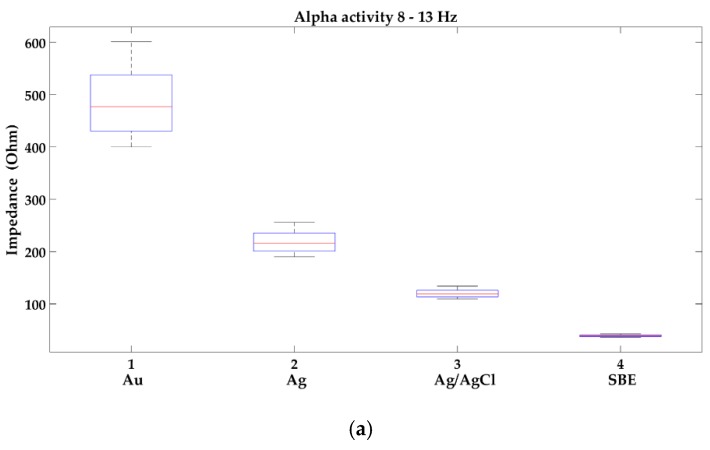

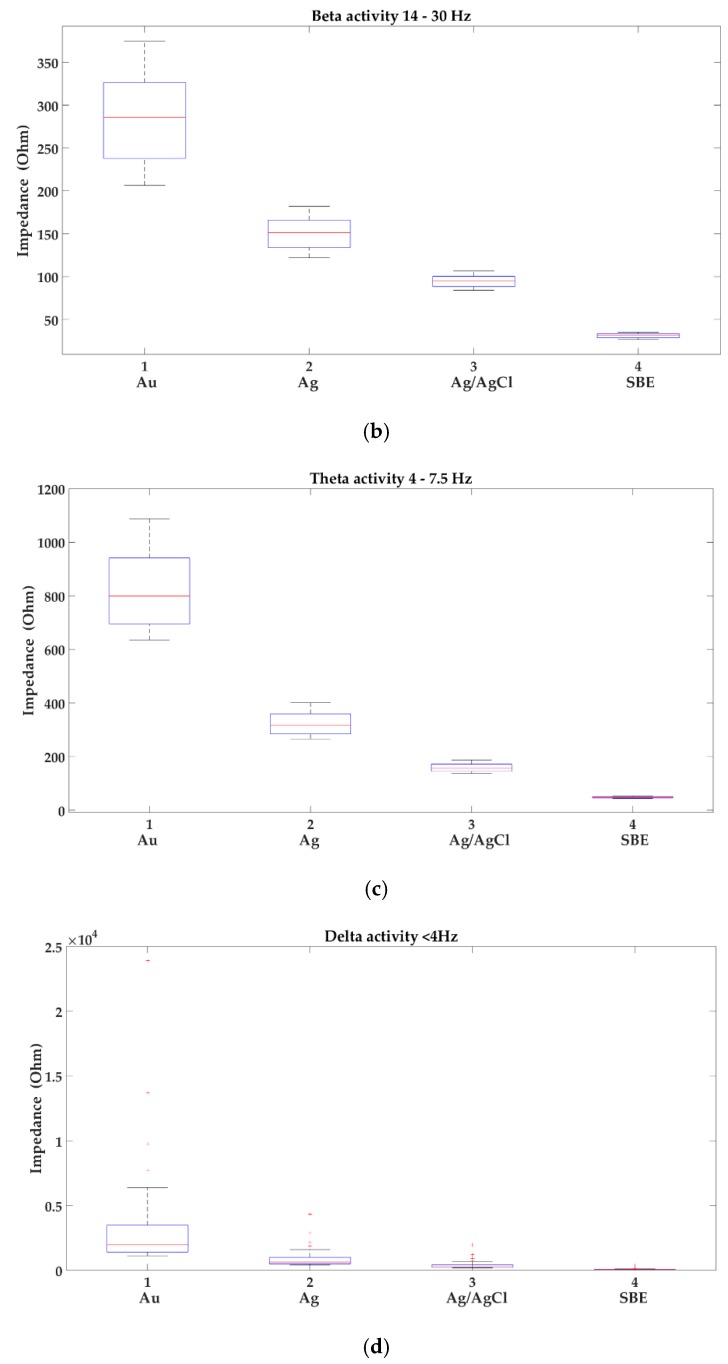
Analysis of the variability of impedance of new electrodes in the following brain activities: (**a**) alpha 8–13 Hz; (**b**) beta 14–30 Hz; (**c**) theta 4–7.5 Hz; (**d**) delta 0.1–3.5 Hz; where: 1-gold (Au), 2-silver (Ag), 3-silver/silver chloride (Ag/AgCl) and 4-sintered bridge electrodes (SBE). Within the box, a horizontal red line means the median value of the data set, whereas the bottom and top blue lines of the box indicate the 25th and 75th percentiles, the whiskers present min and max, and the outliers are marked with the ‘+’ symbol.

In Figure 3 the analysis of changes in the impedance values of new electrodes in selected brain wave ranges, i.e., alpha 8–13 Hz, beta 14–30 Hz, theta 4–7.5 Hz, and delta 0.1–3.5 Hz is shown.

In Figure 3 the occurrence of the variability of impedance is presented. In the range of slow waves, a small impedance variability can be observed in all groups of the new electrodes, but outliers appeared, which occurred with up to 40% of the electrodes from the group: gold, silver, and silver/silver chloride. In turn, in the range of beta waves, impedance variability was the highest in each group. It is worth noting, that the sintered bridge electrodes showed the smallest variability of impedance. Gold electrodes not only have the highest impedance in the entire EEG frequency range but also are characterized by its greatest variability. 

**Figure 4:**
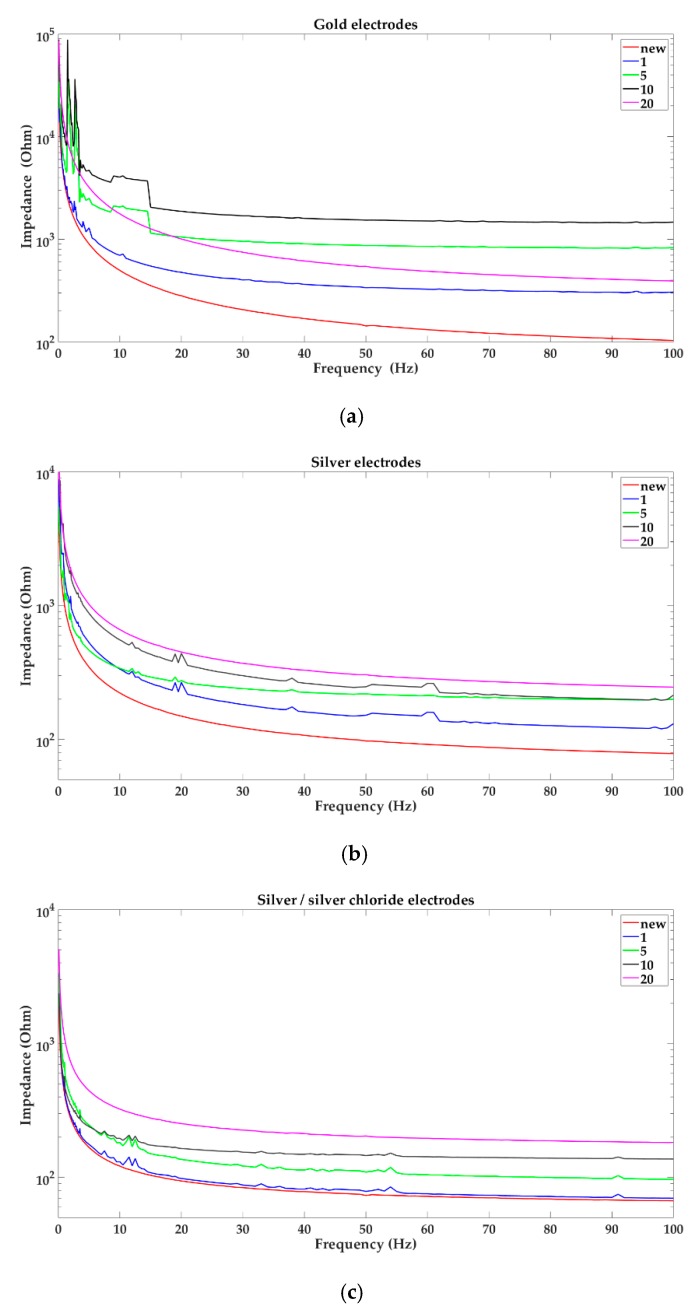

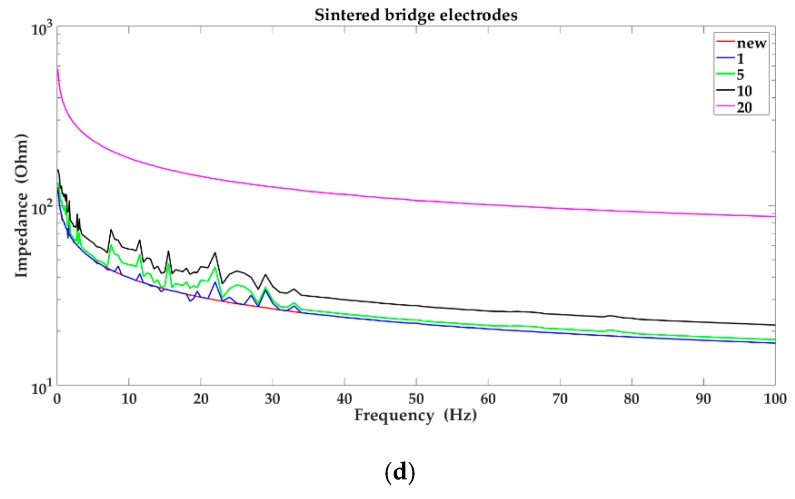
Impedance characteristic of the electrodes: (**a**) gold; (**b**) silver; (**c**) silver/silver chloride; (**d**) sintered bridge electrodes; before the first use, after the first, the fifth, the tenth and the twentieth EEG examinations, respectively.

**Figure 5:**
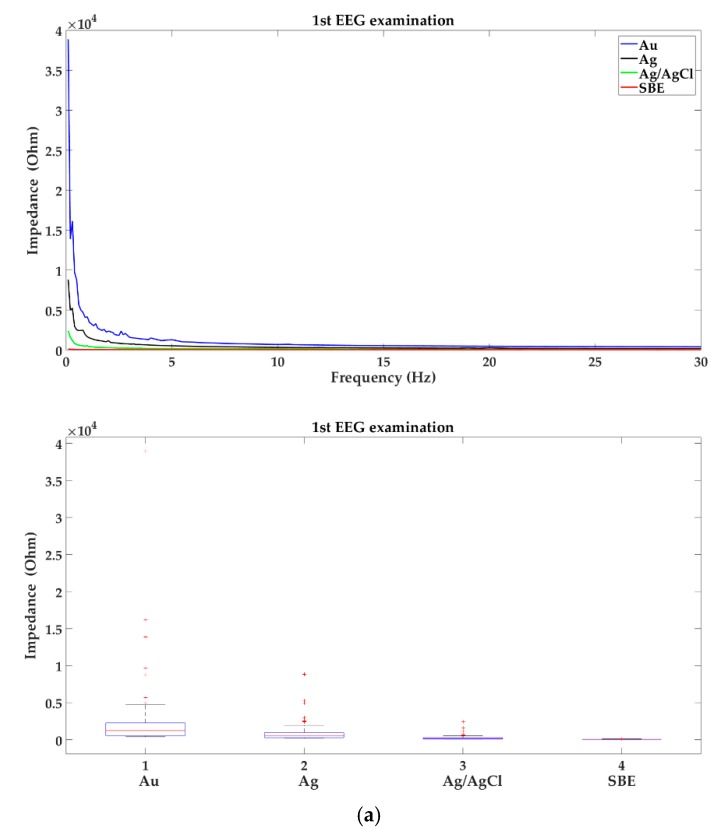

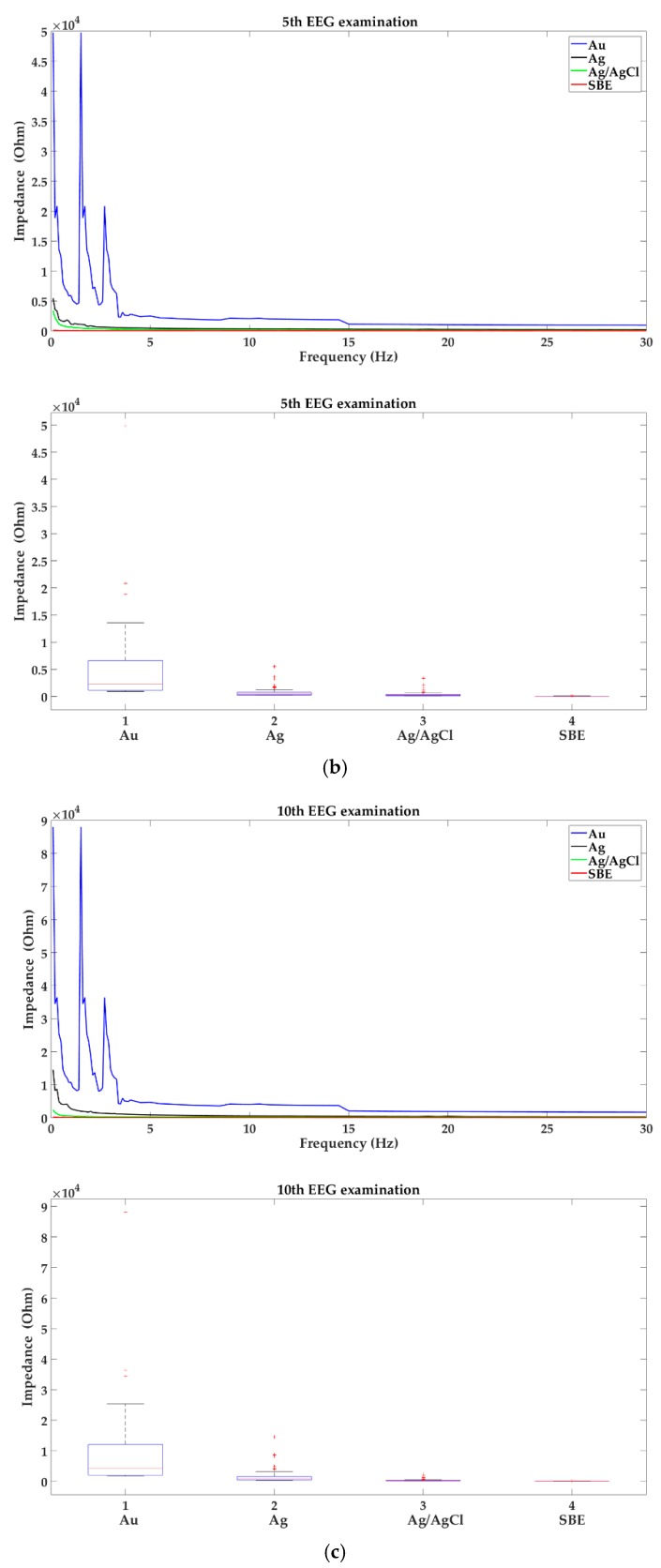

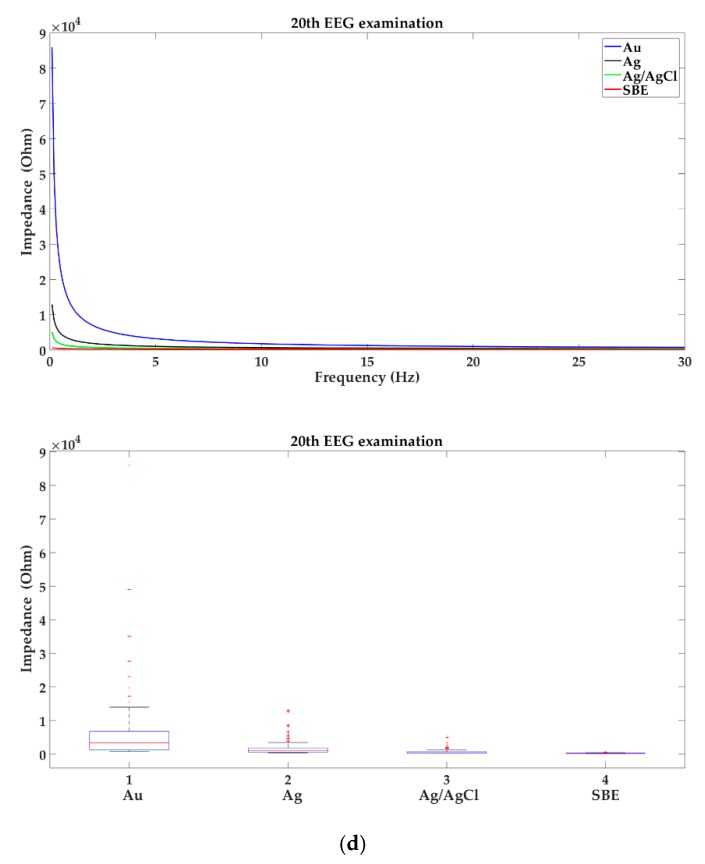
Impedance characteristic of the electrodes: gold (Au), silver (Ag), silver/silver chloride (Ag/AgCl) and sintered bridge electrodes (SBE) in the brain activities 0.1–30 Hz after (**a**) the first; (**b**) the fifth; (**c**) the tenth; and (**d**) the twentieth EEG examinations with analysis of the variability of impedance. Within the box, a horizontal red line means the median value of the data set; whereas the bottom and top blue lines of the box indicate the 25th and 75th percentiles, the whiskers present min and max and the outliers are marked with the ‘+’ symbol.

In Figure 4 and Figure 5, the impedance characteristic of all tested types of electrodes before the first use, after the first, the fifth, the tenth, and the twentieth EEG examinations are presented.

Based on the results presented in Figure 4 and Figure 5, it can be concluded that impedance value and variability of impedance depend on the number of performed EEG examinations. In case of the sintered bridge electrodes, impedance increased with an increase in the number of performed EEG examinations. A similar effect was observed with silver and silver/silver chloride electrodes where the impedance has increased successively after the first, the fifth, the tenth, and the twentieth EEG examinations. For gold electrodes, impedance has increased successively after the first, the fifth, and the tenth EEG examinations. However, after the twentieth EEG examination the impedance approached characteristics corresponding to the first use. Moreover, the variability of impedance increased with a rise in the number of performed EEG examinations for all types of electrodes.

## 4. Discussion 

In clinical practice, it is assumed that the use of gold or silver/silver chloride electrodes, proper preparation of the patient’s scalp, and cleanliness of electrodes should be sufficient to obtain a good quality of EEG data [14,15]. However, our research has shown the existence of dependence of electrode impedance values on the number of performed EEG examinations. This problem seems to be important not only in clinical EEG examination but also in case of the no-clinical EEG-based studies, such as neuroscientific disciplines where reusable electrodes are usually employed [16,17,18,19]. Although the tests were conducted on only ten electrodes of each type, we indicated the permissible number of performed EEG examinations. It should be noticed that the scalp–electrode impedance measured with the EEG equipment for the new and the used electrodes was below 5 kΩ. However, the use of EIS showed significant changes in the values of the electrode–electrolyte–electrode impedance of individual electrodes. In the case of silver/silver chloride electrodes the impedance is acceptable up to the twentieth EEG examination while for gold electrodes it was only up to the tenth use. What is more, the impedance of gold electrodes increased after the tenth EEG examination, and after the twentieth use, the impedance curve approached the course corresponding to the one obtained after the first use. It is supposed that this is due to the electrode coating degradation caused by cleaning. All of the tested gold electrodes are in fact silver electrodes with a 3 μm thick 24 k gold coating. The other electrodes used in EEG studies that are not recommended, i.e., silver electrodes and sintered bridge electrodes, also need to be replaced after 20 uses.

In some cases, i.e., when brain death should be confirmed by EEG examination, it is recommended to obtain not only a scalp–electrode impedance below 5 kΩ but also its equal value for individual electrodes used in the study [20]. In no-clinical applications, e.g., adaptive automation, affective computing, or video games where the human psychophysiological state evaluation is measured by means of EEG equipment [16,17,18,19], the problem with obtaining the same value of impedance for electrodes especially in theta and alpha brain activities should be important as well. The usage of reusable electrodes for measurement of chosen EEG features defining the actual user’s mental status [17,18,19], i.e., ratio of rhythms beta/(alpha + theta), beta/alpha, event related potential, evoked potentials, or the frontal EEG asymmetry may affect the correctness of implementation of all passive brain–computer interface (pBCI) applications. The maintenance of a correct and equal value of the scalp–electrode impedance can be influenced not only by the patient’s preparation for the examination, the skin properties of the subject, and the number of EEG examinations performed with a given set of electrodes but also the variability of impedance values of individual electrodes in one set. The tests carried out with the use of EIS revealed the variability of the impedance in each group of electrodes being tested, i.e., gold, silver, silver/silver chloride, and the sintered bridge electrodes. It is worth noting that the tested electrodes have not been unused. In case of recommended silver/silver chloride electrodes, the largest spread of impedance was observed in range of beta waves, while in range of delta activity, despite small dispersion, outliers appeared. In turn, the gold electrodes achieved the highest impedance and the greatest variability in the entire EEG frequency range. 

On the basis of the obtained results, it can be concluded that the smallest impedance in the EEG frequency range and the smallest spread of impedance values has been obtained for electrodes not recommended for use in EEG examination, i.e., the sintered bridge electrodes. These electrodes are not recommended for several reasons although they are still used. The use of these electrodes requires prior placement in a solution of 0.9% NaCl for 24 h, which limits the number of tests performed to one patient per day. The electrodes are covered with the conducting gel, which has to be supplemented during the EEG examination. Additionally, after each use, the material that covers the electrode disc has to be changed. Those additional operations are inconvenient, but they contribute to the obtaining of the lowest impedance amongst all types of electrodes.

The solution to the problem of the influence of the number of performed EEG examinations on the impedance of electrodes and the occurrence of the variability of impedance may be to propose a new material with appropriate electrochemical properties or the use of the selected electrodes presented in [8,9,10,11,12,13].

In our research we concentrated on the application of reusable electrodes only in clinical EEG examination. The greatest limitations of the presented results were the few experimental observations and the testing of only ten electrodes of each type. For these reasons, in the future, we plan to increase the number of electrodes and experimental observations. The purpose of our further research will be to check the impact of the duration of EEG signal recording on the impedance of electrodes during their repeated use, which could be important in the case of the no-clinical EEG-based studies. Additionally, we will check the existence of the impact of repeated use of one set of electrodes on the amplitude and the asymmetry of the EEG data.

## 5. Conclusions

This paper presents results of impedance analysis of new and used EEG electrodes obtained using the Electrochemical Impedance Spectroscopy method. First, the dependence of the impedance of new electrodes on frequency changes was checked, thus confirming that the impedance of the electrodes decreases with an increase in frequency. During the analysis, occurrence of the variability of impedance in particular groups of new electrodes was additionally observed. The measurements of used electrodes conducted allowed to confirm the dependence of electrode impedance values on the number of performed EEG examinations. The presented research shows that in the case of gold electrodes the permissible number of EEG examinations performed with one set of electrodes is between five and ten, while for other electrodes, i.e., silver electrodes, silver/silver chloride electrodes, and sintered bridge electrodes is between ten and twenty.
